# The role of the Flb protein family in the life cycle of *Aspergillus niger*

**DOI:** 10.1007/s10482-024-01957-x

**Published:** 2024-03-19

**Authors:** Xiaoyi Chen, Juan P. Moran Torres, Han A. B. Wösten

**Affiliations:** Microbiology, Department of Biology, Utrecht University, Padualaan 8, 3584 CH Utrecht, The Netherlands

**Keywords:** Fungus, *Aspergillus*, Asexual development, Flb, Protein secretion

## Abstract

Genes *flbA-E* are involved in sporulation and vegetative growth in *Aspergillus nidulans.* Inactivation of either of these genes results in a fluffy phenotype with delayed or even abolished sporulation. Previously, a non-sporulating phenotype was obtained by inactivating *flbA* in *Aspergillus niger,* which was accompanied by lysis, thinner cell walls, and an increased secretome complexity. Here, we further studied the role of the *flb* genes of *A. niger*. Strains Δ*flbA,* Δ*flbB* and Δ*flbE* showed increased biomass formation, while inactivation of *flbA-D* reduced, or even abolished, formation of conidia. Strain Δ*flbA* was more sensitive to H_2_O_2_, DTT, and the cell wall integrity stress compounds SDS and Congo Red (CR). Also, Δ*flbC* was more sensitive to SDS, while Δ*flbB*, Δ*flbD*, and Δ*flbE* were more sensitive to CR. On the other hand, inactivation of *flbE* increased resistance to H_2_O_2_. Enzyme secretion was impacted when the Δ*flb* strains were grown on xylose. Strain Δ*flbE* showed reduced xylanase, cellulase and amylase secretion. On the other hand, amylase secretion at the periphery of the Δ*flbA* colony was reduced but not in its center, while secretion of this enzyme was increased in the center of the Δ*flbB* colony but not at its periphery. Inactivation of *flbC* and *flbD* also impacted zonal cellulase and amylase activity. Together, the Flb protein family of *A. niger* function in biomass formation, sporulation, stress response, and protein secretion.

**Supplementary Information:**

The online version contains supplementary material available at 10.1007/s10482-024-01957-x.

## Introduction

The FlbA-E proteins of *Aspergillus nidulans* play a role in asexual sporulation. Inactivation of their encoding genes results in fluffy colonies due to the production of high numbers of aerial hyphae combined with the delay or even abolished production of spore producing conidiophores (Wieser et al. 1994).

FlbA is a RGS domain protein, which negatively regulates vegetative growth signaling and thereby stimulates asexual development. It does so by stimulating the intrinsic GTPase activity of the Gα subunit FadA (Yu et al. 1996). Overexpression of *flbA* in *A. nidulans* inhibits hyphal growth and stimulates conidiophore development (Lee and Adams 1994a). By contrast, inactivation of *flbA* results in abolished asexual development (Wieser et al. 1994) and in autolysis of hyphae when colonies mature (Lee and Adams 1994a; Wieser et al. 1994).

Genes *flbB, flbC,* and *flbD* encode transcription factors. FlbB is a fungal specific bZIP-type transcription factor (Etxebeste et al. 2008). Deletion of its encoding gene results in defective branching patterns, in susceptibility to autolysis under high sorbitol or sucrose concentrations and in delayed conidiation with a fluffy appearance. Moreover, mis-scheduled *flbB* upregulation reduces the diameter of the vesicle of the conidiophore and reduces the number of metulae (Etxebeste et al. 2009). FlbC is a transcription factor containing two C_2_H_2_ zinc finger DNA binding domains (Kwon et al. 2010a). Inactivation of its encoding gene results in delayed and reduced conidiation and in enhanced sexual fruiting body formation, while its overexpression restricts hyphal growth and delays conidiation. FlbC acts as a transcriptional regulator in a pathway parallel to that involving FlbA and the FlbB/FlbE and FlbB/FlbD complexes (Garzia et al. 2010). Double mutants cause additive effects, resulting in a prolonged delay in conidiation (Kwon et al. 2010b). Deletion of the c-Myb transcription factor gene *flbD* results in delayed conidiation and a fluffy phenotype (Wieser et al. 1994; Wieser and Adams 1995), while its overexpression causes sporulation in liquid submerged cultures. FlbD functions by interacting with FlbB (Garzia et al. 2009, 2010; Etxebeste et al. 2010).

FlbE does not have any known conserved domain (Garzia et al. 2009). Both inactivation and overexpression of its encoding gene results in the absence of conidiophore formation, accelerated vegetative growth, and accelerated autolysis and cell death (Kwon et al. 2010b). FlbE is involved in FlbB stability and may thus protect FlbB from proteolytic degradation, possibly due to their physical interaction (Garzia et al. 2009). The FlbB/FlbE complex is a prerequisite for *flbD* expression in the wild-type (Garzia et al. 2010).

The Flb proteins are conserved in *A. nidulans, Aspergillus fumigatus, Aspergillus oryzae,* and *Aspergillus niger* (Pel et al. 2007; Ogawa et al. 2010; Kwon et al. 2010a). The phenotypic changes in the Δ*flb* strains of *A. oryzae* are similar to those in *A. nidulans*. Conservation of function of the Flb proteins is also implied by the finding that inactivation of *flbB* in *A. fumigatus* results in delayed and reduced sporulation and precocious cell death (Xiao et al. 2010). Also, the *A. fumigatus flbE* gene is involved in conidiation (Kwon et al. 2010b). However, its inactivation does not result in increased vegetative proliferation, accelerated autolysis, or cell death.

Like in *A. nidulans,* inactivation of *flbA* in *A. niger* results in a fluffy phenotype with abolished sporulation and an increased lysis incidence (Krijgsheld et al. 2013). The latter is probably caused by a reduced thickness (Krijgsheld et al. 2013) and reduced integrity (van Munster et al. 2015) of the cell wall. The Δ*flbA* strain secretes a higher diversity and amount of proteins in the culture medium. From this and the fact that *A. niger* does not secrete proteins in zones that sporulate it was concluded that sporulation inhibits protein secretion in *A. niger*. The fact that inactivation of *flbC* in *A. oryzae* results in reduced expression of the glucoamylase gene *glaB* and the acid protease *pepA* indicates that this sporulation gene also has a positive impact on secretion (Tanaka et al. 2016).

We here assessed the role of the FlbA-E proteins of *A. niger* in sporulation, vegetative growth, secretion, and stress responses. To this end, *flbA* (*ATCC64974_61450*), *flbB* (*ATCC64974_29960*), *flbC* (*ATCC64974_58210*), *flbD* (*ATCC64974_19410*), and *flbE* (*ATCC64974_100850*) were inactivated by CRISPR Cas9. All Flb proteins were shown to impact stress resistance and protein secretion. FlbA-D also play a role in formation of conidia, while FlbA, FlbB and FlbE suppress biomass formation.

## Materials and methods

### Strains and culture conditions

*Escherichia coli* TOP10 was used for cloning. Static and liquid cultures of *A. niger* MA234.1, its derived strains Δ*flbA,* Δ*flbB*, Δ*flbC*, Δ*flbD,* Δ*flbE,* and the strains in which the *flb* genes had been reintroduced (Table 1) were inoculated with spores and grown at 30 ℃. Spores were isolated from 3-day-old cultures that had been grown on potato dextrose agar (PDA) after confluent inoculation with 10^6^ spores. The spores were harvested with 0.9% NaCl using a cotton swab. Spore suspensions were filtered through a syringe with cotton to remove hyphae and counted using a hemocytometer.

**Table 1:** Strains used in this work

*Aspergillus niger*	Genotype	Reference
MA234.1	Δ*akuB, ΔkusA::DR-amdS-DR*	Park et al. 2016
Δ*flbA*	Δ*flbA,* Δ*akuB, ΔkusA::DR-amdS-DR*	This study
Δ*flbB*	Δ*flbB,* Δ*akuB, ΔkusA::DR-amdS-DR*	This study
Δ*flbC*	Δ*flbC,* Δ*akuB, ΔkusA::DR-amdS-DR*	This study
Δ*flbD*	Δ*flbD,* Δ*akuB, ΔkusA::DR-amdS-DR*	This study
Δ*flbE*	Δ*flbE,* Δ*akuB, ΔkusA::DR-amdS-DR*	This study
Δ*flbA:: flbA*^+^	Δ*flbA,* Δ*akuB, ΔkusA::DR-amdS-DR flbA*^+^	This study
Δ*flbB:: flbB*^+^	Δ*flbB,* Δ*akuB, ΔkusA::DR-amdS-DR flbB*^+^	This study
Δ*flbC:: flbC*^+^	Δ*flbC,* Δ*akuB, ΔkusA::DR-amdS-DR flbC*^+^	This study
Δ*flbD:: flbD*^+^	Δ*flbD,* Δ*akuB, ΔkusA::DR-amdS-DR flbD*^+^	This study
Δ*flbE:: flbE*^+^	Δ*flbE,* Δ*akuB, ΔkusA::DR-amdS-DR flbE*^+^	This study

For static cultures, 10^6^ spores were point inoculated on minimal medium (MM; 70.6 mM NaNO_3_, 11 mM KH_2_PO_4_, 6.7 mM KCl, 2 mM MgSO_4_.7H_2_O, and trace elements solution (Vishniac and Santer 1957)) with 1% glucose and 1.5% agar (MMA-G). Glucose was replaced with 1% (w/v) pectin, sucrose, xylose, xylan, starch, maltose, or sorbitol to assess sporulation on these carbon sources. Phenotyping of strains was also done on PDA.

Colonies were grown in between two perforated polycarbonate membranes (pores of 0.1 µm, diameter 76 mm; Profiltra, Almere, The Netherlands) (Wösten et al. 1991) on MMA-G for biomass assessment of static cultures. The upper polycarbonate membrane was placed 24 h after inoculation. To monitor spatial protein secretion, 7-day-old colonies that had been grown on a single PC membrane were transferred for 24 h to a ring plate (Levin et al. 2007). The five concentric wells of this plate were filled with MM with 25 mM xylose (MM-X).

### Gene inactivation constructs

Three plasmids were constructed for inactivation of each of the genes *flbA, flbB*, *flbC* and *flbD*. Two of these plasmids were made to express a sgRNA targeting either the 5’ or the 3’ end of the coding sequence of the target gene, while one construct was made in which flanking sequences of the target gene were cloned (Supplemental Fig. 1A). In the case of *flbE*, only one sgRNA construct was made because of the small size of this gene. The 23 bp sgRNAs were selected using CHOPCHOP (https://chopchop.cbu.uib.no/) and cloned between the proline tRNA promoter (ptRNA-pro1) and terminator (tracrRNA::term) using *PacI* linearized pFC332 (Nodvig et al. 2015). To this end, the promoter was amplified from plasmid pTLL108.1 (van Leeuwe et al. 2019) using primer pairs 1/3 (*flbA*sgRNA1), 1/4 (*flbA*sgRNA2), 1/5 (*flbB*sgRNA1), 1/6 (*flbB*sgRNA2*)*, 1/7 (*flbC*sgRNA1), 1/8 (*flbC*sgRNA2), 1/9 (*flbD*sgRNA1), 1/10 (*flbD*sgRNA2) and 1/11 (*flbE*sgRNA) (Supplemental Table 1). The terminator was amplified from plasmid pTLL109.2 (van Leeuwe et al. 2019) by using primer pairs 2/12 (*flbA*sgRNA1), 2/13 (*flbA*sgRNA2), 2/14 (*flbB*sgRNA1), 2/15 (*flbB*sgRNA2), 2/16 (*flbC*sgRNA1), 2/17 (*flbC*sgRNA2), 2/18 (*flbD*sgRNA1), 2/19 (*flbD*sgRNA2) and 2/20 (*flbE*sgRNA) (Supplemental Table 1). The promoter, terminator, and sgRNA sequences were assembled using NEBuilder (New England Biolabs, international.neb.com) resulting in plasmids pFC332-sgRNA1-*flbA*, pFC332-sgRNA2-*flbA,* pFC332-sgRNA1-*flbB*, pFC332-sgRNA2-*flbB,* pFC332-sgRNA1-*flbC*, pFC332-sgRNA2-*flbC,* pFC332-sgRNA1-*flbD*, pFC332-sgRNA2-*flbD* and pFC332-sgRNA1-*flbE*.

**Fig. 1:**
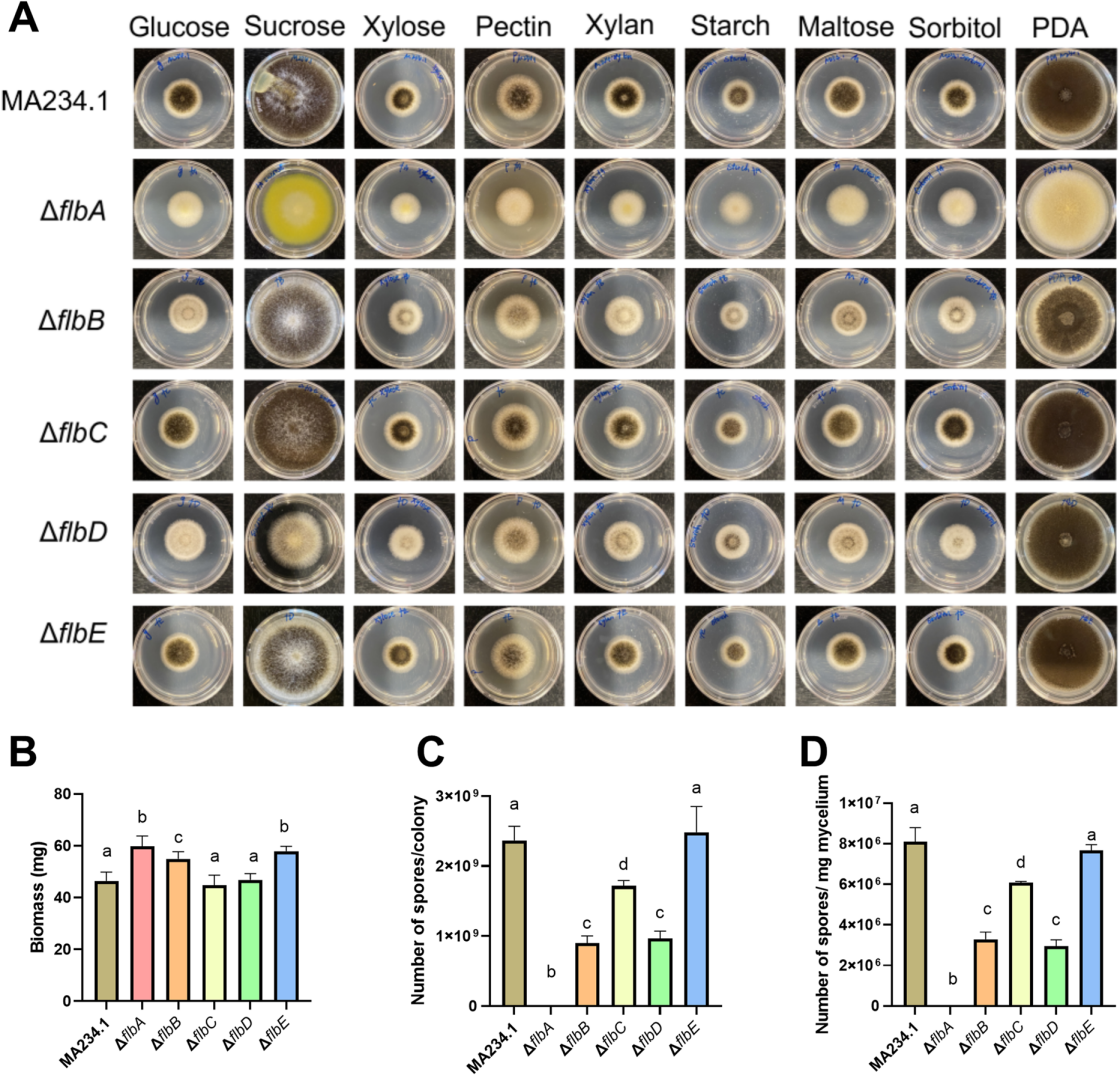
Growth of *A. niger* MA234.1 and the ∆*flbA*, ∆*flbB*, ∆*flbC*, ∆*flbD* and ∆*flbE* strains on PDA and on MMA with different carbon sources (**A**), as well as biomass (**B**), number of spores (**C**), and number of spores per mg mycelium (**D**) of cultures grown on MMA with glucose (MMA-G). Cultures were grown for 5 (A) and 7 (B) days from a point inoculum or grown for 3 days after confluent spreading of spores on a plate (C, D). To determine biomass, colonies were grown in between two perforated polycarbonate membranes, while spores were harvested from colonies that had grown on MMA-G in the absence of the membranes. Statistical analysis was done with One-way ANOVA with different letters indicating statistical differences

The upstream–downstream fragments of the target genes were amplified from genomic DNA using primer pairs 21/22 (upstream *flbA*), 23/24 (upstream *flbB*), 25/26 (upstream *flbC*), 27/28 (upstream *flbD*), 29/30 (upstream *flbE*), 31/32 (downstream *flbA*), 33/34 (downstream *flbB*), 35/36 (downstream *flbC*), 37/38 (downstream *flbD*) and 39/40 (downstream *flbE*) (Supplemental Table 1). The up- and down-stream sequences of each gene were introduced in pUC19 (primer pair 96/97) using NEBuilder, yielding plasmids pUC19-*flbA*, pUC19-*flbB*, pUC19-*flbC*, pUC19-*flbD*, and pUC19-*flbE* (Supplemental Fig. 1B).

### Constructs for reintroduction of the flb genes

Two plasmids were constructed to reintroduce each of the genes *flbA*, *flbB*, *flbC, flbD* and *flbE*. One construct was made to express a sgRNA targeting either the 3’ end of the promoter or the 5’ end of the terminator of the target gene, while one construct was made in which flanking and coding sequences of this gene were cloned (Supplemental Fig. 2A). The 23 bp sgRNAs were selected using CHOPCHOP (https://chopchop.cbu.uib.no/) and cloned between the proline tRNA promoter (ptRNA-pro1) and terminator (tracrRNA::term) using *PacI* linearized pFC332 (Nodvig et al. 2015). To this end, the ptRNA-pro1 promoter was amplified from plasmid pTLL108.1 using primer pairs 1/66 (*flbA*), 1/67 (*flbB*), 1/68 (*flbC*), 1/69 (*flbD*) and 1/70 (*flbE*) (Supplemental Table 1), while the terminator was amplified from pTLL109.2 using primer pairs 71/2 (*flbA*), 72/2 (*flbB*), 73/2 (*flbC*), 74/2 (*flbD*) and 75/2 (*flbE*) (Supplemental Table 1). This resulted in plasmids pFC332-sgRNA-*flbA*-com, pFC332-sgRNA-*flbB*-com, pFC332-sgRNA-*flbC*-com, pFC332-sgRNA-*flbD*-com and pFC332-sgRNA-*flbE*-com (Supplemental Fig. 2A).

**Fig. 2:**
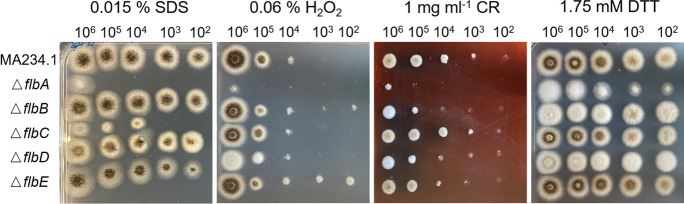
Resistance of the reference and the Δ*flbA*, Δ*flbB*, Δ*flbC,* Δ*flbD,* and Δ*flbE* strains to 0.015% SDS, 1.75 mM DTT, 1 mg mL^−1^ CR, and 0.06% H_2_O_2_ after 5 days of growth on MMA-G

The 5’ and 3’ flanks as well as the coding sequences of *flbA*, *flbB*, *flbC*, *flbD*, and *flbE* were amplified from genomic DNA by PCR using primer pairs 21/51 (*flbA* 5’ flank), 31/32 (*flbA* 3’ flank), 56/57 (gene *flbA*); 23/52 (*flbB* 5’ flank), 33/34 (*flbB* 3’ flank), 58/59 (gene *flbB*); 25/53 (*flbC* 5’ flank), 35/36 (*flbC* 3’ flank), 60/61 (gene *flbC*); 27/54 (*flbD* 5’ flank), 37/38 (*flbD* 3’ flank), 62/63 (gene *flbD*); and 29/55 (*flbE* 5’ flank), 39/40 (*flbE* 3’ flank), 64/65 (gene *flbE*) (Supplemental Table 1). This resulted in plasmids pUC19-*flbA*-com, pUC19-*flbB*-com, pUC19-*flbC*-com, pUC19-*flbD*-com and pUC19-*flbE*-com (Supplemental Fig. 2B).

### Transformation of A. niger

Transformation of *A. niger* was done as described (de Bekker et al. 2009). Mycelium was protoplasted that was grown for 16 h in liquid shaken cultures in TM-G (MM with 0.5% yeast extract, 0.2% casamino acids and 25 mM glucose as a carbon source). Gene deletion was performed by co-transforming the three (*flbA-D*) or two (*flbE*) plasmids for each gene (see above). Transformants were selected on MMA-S (MM medium with 2 M sucrose and 1.5% agar) with 150 μg mL^−1^ hygromycin, purified twice on MMA-G with 150 μg mL^−1^ hygromycin, and transferred to PDA without antibiotic. After 2 days, the colonies were transferred to MMA-G with or without 150 μg mL^−1^ hygromycin to confirm that the two (*flbA-D*) or one (*flbE*) sgRNA constructs that contain a hygromycin resistance cassette were lost in the transformant. Gene deletion was confirmed by PCR (Supplemental Fig. 1C) using primer pairs 41/42 (*flbA*), 43/44 (*flbB*), 45/46 (*flbC*), 47/48 (*flbD*) and 49/50 (*flbE*) (Supplemental Table 1). The resulting fragments were sequenced (Macrogen, www.macrogen-europe.com).

Hygromycin was also used for selecting transformants in which the wild-type *flb* gene was reintroduced. These strains were obtained by co-transforming the two constructs made for reintroduction for each of the genes (see above). Reintroduction was confirmed by PCR (Supplemental Fig. 2C) using primer pairs 76/77 and 78/79 (*flbA*), 80/81 and 82/83 (*flbB*), 84/85 and 86/87 (*flbC*), 88/89 and 90/91 (*flbD*), 92/93 and 94/95 (*flbE*) (Supplemental Table 1). The resulting fragments were sequenced (Macrogen, www.macrogen-europe.com).

### SDS-PAGE

Proteins contained in 400 μL spent culture medium were precipitated overnight in 4 volumes pre-cooled acetone at -20 °C, collected at 4 °C at 20,000 g for 2 min and dissolved in 20 μL loading buffer (20% glycerol, 4% SDS, 100 mM Tris–HCl pH 6.8, 0.01% bromophenol blue). Composition and running of the SDS-PAA gels was done as described (Lyu et al. 2023).

### Enzyme activity assays

Cellulase activity was measured using the filter paper activity assay (Fpase) (Xiao et al. 2004). To this end, 7 mm diameter circles of Whatman No.1 filter paper were placed in 96 well plates with 60 µl culture medium for 24 h at 50 °C, followed by a 5 min incubation at 95 °C after adding 120 µl DNS (10 g L^−1^ 3,5-dinitrosalicylic acid, 400 g L^−1^ KNa-tartrate and 16 g L^−1^ NaOH). Samples (100 µl) were transferred to the wells of a 96 wells flat-bottom plate (Cellstar, Greiner Bio-one, www.gbo.com) and the A_540_ was determined using a Synergy HTX Microplate Reader (BioTek, www.agilent.com). Activity was determined using a glucose standard curve. A unit of cellulase activity was defined as 1 µmol glucose released in 1 min. Amylase activity was determined in a similar way as cellulase activity but the Whatman filter paper was replaced by 60 μl 1% starch. Xylanase activity was determined using the Xylanase Assay Kit (XylX6 Method)(Megazyme, www.megazyme.com). In this case, one unit of activity was defined as the amount of enzyme required to release 1 µmole of 4-nitrophenol from the XylX6 substrate in one minute under the defined assay conditions.

### Statistics

Experiments were performed using biological triplicates. Data were subjected to One-way Anova analysis of variance. Mean value was analysed with a confidence P ≤ 0.05.

## Results

### Inactivation and reintroduction of flbA-flbE

FlbA-E from *A. niger* show 62.0–79.7% and 66.2–82.6% identity to their homologues of *A. fumigatus* and *A. nidulans,* respectively (NCBI, https://www.ncbi.nlm.nih.gov/). Genes *flbA, flbB, flbC, flbD, and flbE* were inactivated in *A. niger.* Their inactivation was confirmed by PCR (see Material and Methods) and Sanger sequencing. In addition, wild-type phenotypes were obtained after reintroducing the inactivated genes in the deletion strains (data not shown).

### Growth and sporulation

Radial growth of ∆*flbA*, ∆*flbB*, ∆*flbC*, ∆*flbD*, and ∆*flbE* colonies was similar to the reference strain when grown on PDA or on defined MMA with glucose, sucrose, xylose, sorbitol, maltose, starch, xylan or pectin as a carbon source (Fig. 1A). However, strains Δ*flbA,* Δ*flbB,* and Δ*flbE* produced 10.4–20.3% more biomass on MMA-G (Fig. 1B) compared to the reference strain. Spores were counted from cultures grown on MMA-G. The Δ*flbA* strain did not form spores at all, while Δ*flbB,* Δ*flbC,* and Δ*flbD* showed a reduced formation of conidia of 27.4%-62.0% (Fig. 1C, D). Spore formation of Δ*flbE* was similar to that of the reference strain.

### Stress resistance

The reference and the Δ*flb* strains were exposed to the cell wall stressors sodium dodecyl sulfate (SDS) and Congo Red (CR) and the endoplasmic reticulum stressor dithiothreitol (DTT) as well as to H_2_O_2_ induced oxidative stress (Fig. 2). Strain Δ*flbA* showed increased sensitivity to all stressors. Strain Δ*flbC* was more sensitive to SDS, while Δ*flbB*, Δ*flbD* and Δ*flbE* were more sensitive to CR. On the other hand, Δ*flbE* showed higher resistance to H_2_O_2_.

### Protein secretion

**Fig. 3:**
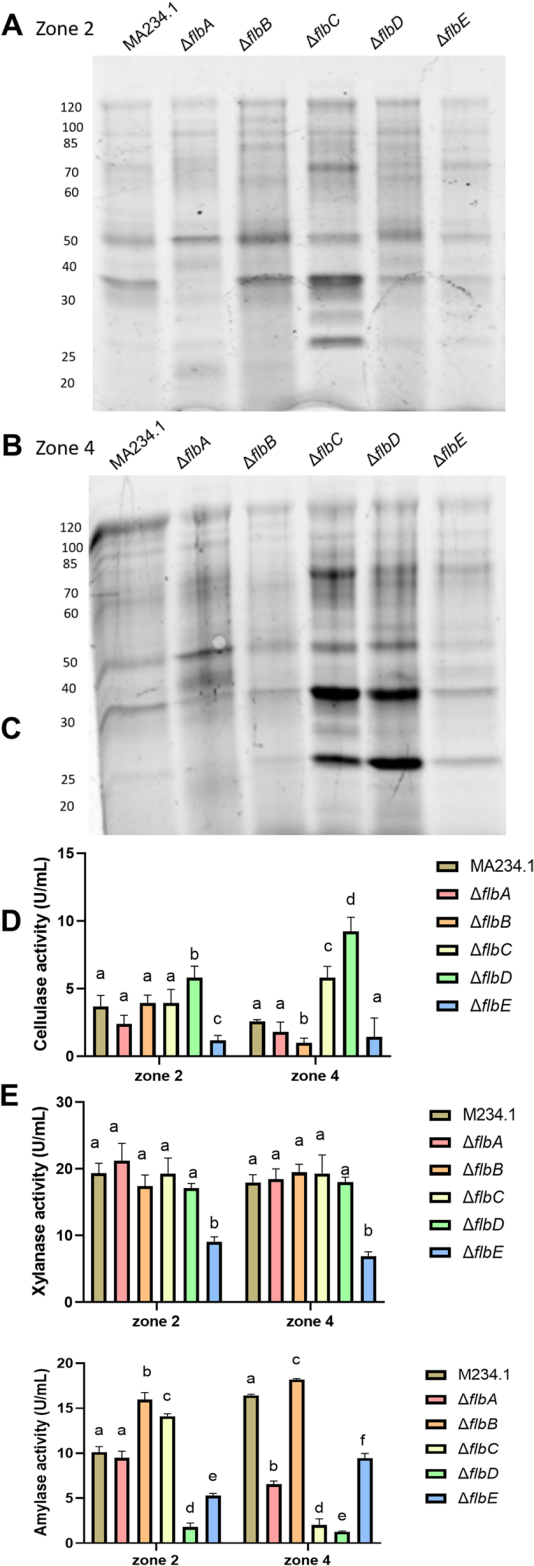
Protein profiles (**A**, **B**) and cellulase (**C**), xylanase (**D**), and amylase (**E**) activity of the reference and the Δ*flb* strains. To monitor spatial protein secretion, 7-day-old colonies that had grown on a single PC membrane were transferred for 24 h to a ring plate (Levin et al. 2007). The five concentric wells of this plate were filled with MM with 25 mM xylose (MM-X). Ring 2 is a central zone, ring 4 is just behind the colony periphery. Statistical analysis was done with One-way ANOVA with different letters indicating statistical differences

Colonies of the reference and the Δ*flb* strains that had been grown for 7 days on a perforated PC membrane (see Material and Methods) were transferred for 24 h to a ring plate with 5 concentric wells (Levin et al. 2007) filled with MM-X. This medium contains xylose, which induces a wide range of xylanolytic and cellulolytic enzymes (van Peij et al. 1998). Also, it inhibits but not abolishes glucoamylase secretion (Nunberg et al. 1984; Wösten et al. 1991). SDS-PAGE showed that protein profiles of the central zone 2 of Δ*flbA* and Δ*flbC* colonies was different from that of the reference strain, while those of the other *flb* strains were not affected (Fig. 3A). Strains Δ*flbA*, Δ*flbC*, and Δ*flbD* indicated higher protein intensity in the sub-periphery zone 4 of the colony when compared to the reference strain, while protein profiles were not affected in Δ*flbB* and Δ*flbE* (Fig. 3A, B).

Activity of cellulase, xylanase, and amylase was determined in the culture medium of the reference and the Δ*flb* strains in zones 2 (centre) and 4 (sub-periphery) of the ring plate (Fig. 3C-E). Xylanase activity was not affected in the Δ*flb* strains except for Δ*flbE* that showed a 2.5-fold (zone 2) and 2.6-fold (zone 4) lower activity (Fig. 3D). Cellulase and amylase activities were also lower in the culture medium of Δ*flbE*. Cellulase activity was 3.1-fold (zone 2) and 1.8-fold (zone 4) lower (Fig. 3C), while amylase activity was 1.9-fold (zone 2) and 1.7-fold (zone 4) lower (Fig. 3E). A decreased amylase activity (8.2-fold) was also found in zone 4 of Δ*flbC,* zone 2 (6.7-fold) and zone 4 (13.1-fold) of Δ*flbD,* and zone 4 (2.5-fold) of Δ*flbA*. On the other hand, amylase was 1.4 fold and 1.6 fold higher in zone 2 of Δ*flbC* and Δ*flbB.* Also, cellulase activity was 2.1-fold and 3.8-fold higher in zone 4 of the Δ*flbC* and Δ*flbD* strains, respectively, when compared to the reference strain (Fig. 3C). Together, the Flb proteins can play both a stimulatory as well as a repressing role in release of enzymes that are involved in substrate degradation.

## Discussion

**Fig. 4:**
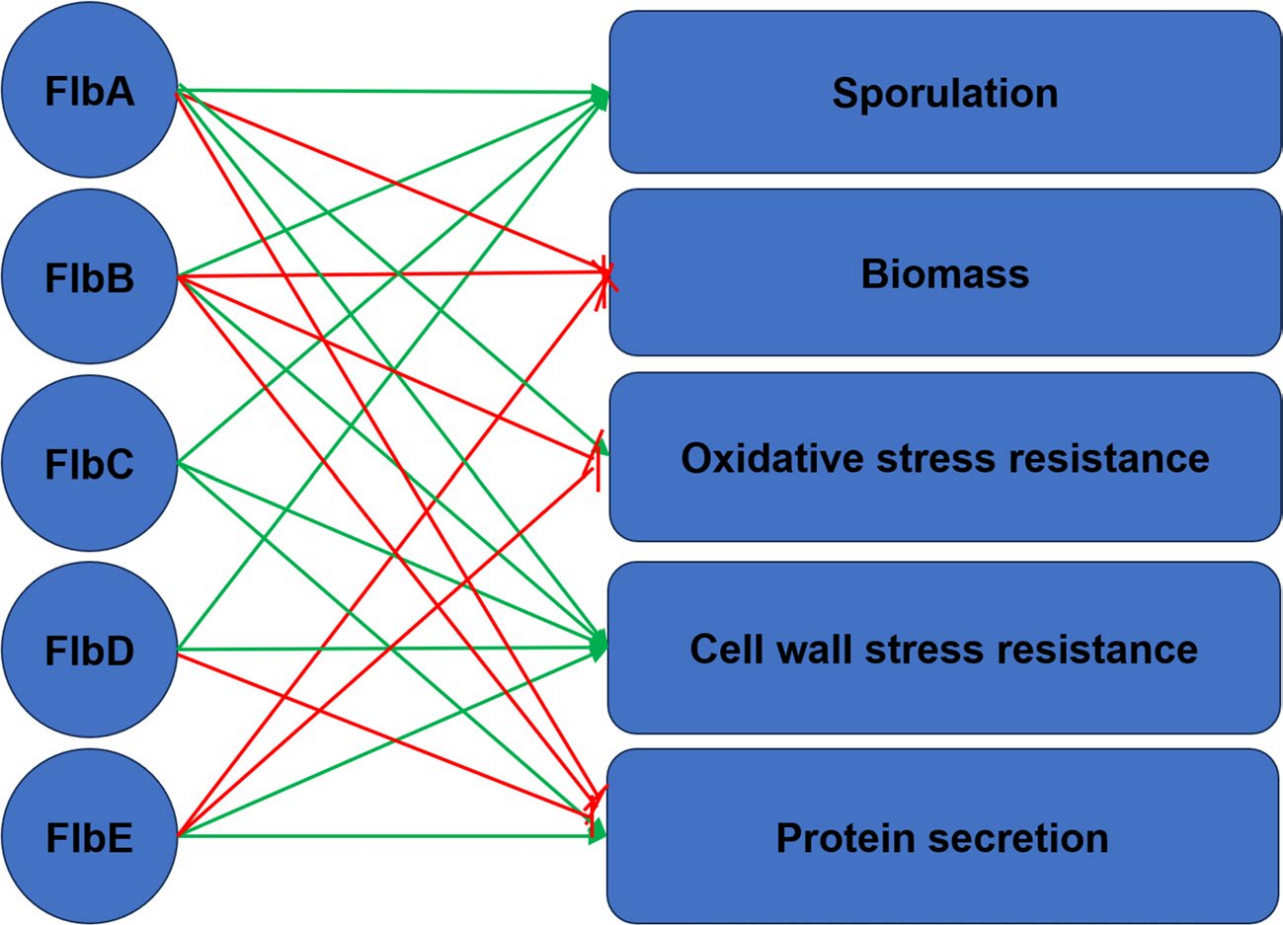
Pleiotropic roles of the Flb proteins of *A. niger*. Red and green lines indicate repression and stimulation, respectively

The Flb proteins of *A. niger* were shown to have roles in biomass formation, sporulation, stress resistance, and secretion. All Flb family proteins play a role in protein secretion and in resistance to cell wall stress. FlbA also functions in resistance to oxidative and ER stress, while FlbE has a negative effect on oxidative stress resistance. In addition, FlbA, FlbB and FlbE have repressive effects on biomass formation, while FlbA-D function in production of conidia. Together, Flb proteins of *A. niger* have pleiotropic phenotypes (Fig. 4).

FlbA, FlbB and FlbE seem to have a conserved role in biomass formation in aspergilli. Overexpression of *flbA* in *A. nidulans* inhibits hyphal growth (Lee and Adams 1994a), while its inactivation results in autolysis of hyphae (Lee and Adams 1994a; Wieser et al. 1994). Deletion of *flbB* in *A. nidulans* results in defective branching patterns and in susceptibility to autolysis when exposed to high osmotic media (Etxebeste et al. 2009), while inactivation of *flbB* in *A. fumigatus* results in precocious cell death (Xiao et al. 2010). Notably, both inactivation and overexpression of *flbE* in *A. nidulans* results in accelerated vegetative growth, autolysis and cell death (Kwon et al. 2010b). This indicates that the level of FlbE within hyphae of *A. nidulans* is important for its function. Whether this is also the case for other aspergilli needs to be established. A role in vegetative growth was also shown for FlbC of *A. nidulans* (Kwon et al. 2010a)*.* This was not observed in our study in *A. niger* but this may be due to the fact that the phenotype in *A. nidulans* was found upon overexpression of *flbC*, while we performed a *flbC* deletion. Similarly, a role of FlbD in hyphal growth has been described in *A. oryzae* (Ogawa et al. 2010) but we did not find it in *A. niger*.

The *flb* genes of *A. nidulans* were originally found by the isolation of fluffy colonies with a delayed or even abolished sporulation (Wieser et al. 1994). Similar sporulation phenotypes were found for all *flb* genes of *A. oryzae* (Ogawa et al. 2010) and for *flbB* and *flbE* of *A. fumigatus* (Xiao et al. 2010; Kwon et al. 2010b). We here showed that *flbA-D* of *A. niger* also play a role in sporulation but this was not the case for *flbE.* Thus, although the *flb* genes seem to have conserved functions in biomass formation and sporulation there are differences between the aspergilli. This was previously also shown for *fluG* of *A. niger* (Wang et al. 2015). FluG is involved in sporulation in *A. nidulans* (Lee and Adams 1994b, 1996) and *A. oryzae* (Ogawa et al. 2010) but not in *A. niger* (Wang et al. 2015) and in air-exposed cultures of *A. fumigatus* (Mah and Yu 2006).

Previously, a relation between sporulation and repression of secretion was found in *A. niger* (Levin et al. 2007; Krijgsheld et al. 2013). Secretion of proteins was only observed in non-sporulating colonies of this fungus (Levin et al. 2007). Secretion was observed throughout the colony after inactivating *flbA*, which is explained by the non-sporulating phenotype (Krijgsheld et al. 2013). By contrast, the Δ*brlA* strain did not show altered secretion. This strain that lacks the central regulator of sporulation initiates but does not complete sporulation. This indicates that the regulatory link between sporulation and secretion occurs upstream of BrlA. The secretome of xylose-grown Δ*flbA* colonies contained 18 proteins with a signal sequence for secretion that had never been reported to be part of the secretome of *A. niger*, while 101 proteins had previously not been identified in the culture medium of xylose-grown wild type colonies (Krijgsheld et al. 2013). From these data it was concluded that inactivation of *flbA* results in spatial changes in secretion and in a more complex secretome. SDS PAGE and enzyme activity assays showed that the other Flb proteins also impact secretion of enzymes by xylose-grown colonies. Inactivation of *flbE* resulted in a reduced xylanase, cellulase and amylase activity in the culture medium underlying the outer and central zones of the colony. Decreased amylase activity was also found in the culture medium underlying the inner and outer zone of Δ*flbD,* as well as in the outer zone of Δ*flbC.* On the other hand, amylase activity was higher in the culture medium underlying the inner zone of Δ*flbC* and Δ*flbB* and cellulase activity was higher in the outer zone of the Δ*flbC* and Δ*flbD* strains. Together, these data show that the Flb proteins of *A. niger* can both stimulate and repress protein activity in the medium. Repression of protein secretion into the culture medium makes sense when a colony initiates sporulation and thereby “decides” to invest in reproduction and not in vegetative growth. The stimulatory role of Flb proteins in this respect is not clear yet. Apart from the Flb proteins, also FluG of *A. niger* seems to play a role in regulation of secretion (Wang et al. 2015). Thus, FluG of *A. niger* has lost its role in sporulation but would still be functional in regulation of secretion.

The Flb proteins of *A. niger* also function in stress resistance. FlbE has a negative effect on resistance to H_2_O_2_. On the other hand, FlbA has a stimulatory role in resistance to the cell wall stressors SDS and Congo Red, as well as to the endoplasmic reticulum stressor DTT and to H_2_O_2_. The reduced resistance of Δ*flbA* to cell wall stressors is explained by a reduced thickness (Krijgsheld et al. 2013), and reduced integrity (van Munster et al. 2015) of its cell wall. The role of FlbA in resistance to H_2_O_2_ and to DTT is less easily explained. Previously it was shown that FlbA downregulates *rpnR* (Aerts et al. 2019). RpnR promotes resistance to H_2_O_2_ and to DTT. The fact that FlbA promotes resistance to these stressors and at the same time represses RpnR is contradictory and may be explained by the complex regulatory pathways of stress resistance. The fact that FlbB-D also protect against one of the cell wall stressors suggests that such a protection may be particular relevant during sporulation.

Together, we here showed that Flb proteins of *A. niger* are not only involved in regulation of vegetative growth and sporulation as was previously shown in *A. nidulans,* but also in regulation of secretion and in stress resistance. The fact that FlbB of *A. nidulans* represses production of the secondary metabolite 2,4-dihydroxy-3-methyl-6-(2-oxopropyl) benzaldehyde (DHMBA) (Oiartzabal-Arano et al. 2015), while FlbC of *A. oryzae* stimulates expression of the glucoamylase gene *glaB* and the acid protease *pepA* (Tanaka et al. 2016) suggests that the Flb proteins of other aspergilli also have pleiotropic phenotypes.

## Supplementary Information

Below is the link to the electronic supplementary material.

Supplementary file1 (DOCX 392 KB)

## Data Availability

No datasets were generated or analysed during the current study.
