# Supplementary material for: The role of the Flb protein family in the life cycle of Aspergillus niger
Source: Antonie Van Leeuwenhoek. 2024 Mar 19;117(1):58. doi: 10.1007/s10482-024-01957-x (PMC10950988; doi:10.1007/s10482-024-01957-x)
Supplement: Supplementary file 1 — Supplementary file1 (DOCX 392 KB) [file 10482_2024_1957_MOESM1_ESM.docx]

**Supplemental Material**

**The role of the Flb protein family in the life cycle of *Aspergillus niger***

Xiaoyi Chen, Juan P. Moran Torres, Han A. B. Wösten

**Supplemental Table 1.** Primers used in this study. Nucleotide sequences overlapping between DNA fragments are indicated by lower case font.

| **Number** | **Primer name** | **Primer sequence (5’-3’)** |  |
| --- | --- | --- | --- |
| 1 | sgRNA universal-F | gtttccgctgagggtttaatACTCCGCCGAACGTACTG |  |
| 2 | sgRNA universal-R | ctgtctcggctgaggtcttaAAAAGCAAAAAAGGAAGGTACAAAAAAGC |  |
| 3 | gRNA1-flbA-P-R | GGGATAGGATCAACAGACGGgacgagcttactcgtttcg |  |
| 4 | gRNA2-flbA-P-R | AGATCTTTTAGTAATCACCAgacgagcttactcgtttcg |  |
| 5 | gRNA1-flbB-P-R | CCGAGATCTCTTGTGTGTATgacgagcttactcgtttcg |  |
| 6 | gRNA2-flbB-P-R | GGAATGGGACGAAGTTTGAGgacgagcttactcgtttcg |  |
| 7 | gRNA1-flbC-P-R | GGAATGGGACGAAGTTTGAGgacgagcttactcgtttcg | |
| 8 | gRNA2-flbC-P-R | TGGGTGTCTTGTATGCTCCAgacgagcttactcgtttcg |  |
| 9 | gRNA1-flbD-P-R | CGATGATCTCGGACCACAACgacgagcttactcgtttcg |  |
| 10 | gRNA2-flbD-P-R | CCAGTTCTTAACCGCATTGTgacgagcttactcgtttcg |  |
| 11 | gRNA-flbE-P-R | GATGACTTTCCCACCGACGTgacgagcttactcgtttcg |  |
| 12 | gRNA1-flbA-T-F | CCGTCTGTTGATCCTATCCCgttttagagctagaaatagcaag |  |
| 13 | gRNA2-flbA-T-F | TGGTGATTACTAAAAGATCTgttttagagctagaaatagcaag |  |
| 14 | gRNA1-flbB-T-F | ATACACACAAGAGATCTCGGgttttagagctagaaatagcaag | |
| 15 | gRNA2-flbB-T-F | TATGGGGTTCAACGCCAGAGgttttagagctagaaatagcaag |  |
| 16 | gRNA1-flbC-T-F | CTCAAACTTCGTCCCATTCCgttttagagctagaaatagcaag |  |
| 17 | gRNA2-flbC-T-F | TGGAGCATACAAGACACCCAgttttagagctagaaatagcaag |  |
| 18 | gRNA1-flbD-T-F | GTTGTGGTCCGAGATCATCGgttttagagctagaaatagcaag |  |
| 19 | gRNA1-flbD-T-F | ACAATGCGGTTAAGAACTGGgttttagagctagaaatagcaag |  |
| 20 | gRNA-flbE-T-F | ACGTCGGTGGGAAAGTCATCgttttagagctagaaatagcaag |  |
| 21 | flbA-up-F | tcgccattcaggctgGAATTCACCCACGGACAGTAGACGGC |  |
| 22 | flbA-up-R | atttccaaaagggagaaagaGGGAAAGGTGGAGGGGTGGGTGT |  |
| 23 | flbB-up-F | tcgccattcaggctgGAATTCACCCACGGACAGTAGACGGC |  |
| 24 | flbB-up-R | tcagtagtagatggccgacggGGGAGGACGGAGGCGGGAAGAA |  |
| 25 | flbC-up-F | tcgccattcaggctgGAATTCACACTGGCGCCGTTTCGAAG |  |
| 26 | flbC-up-R | cacggcgcaaagatatccctggGGGGAATTGCGCGGGTGAACT |  |
| 27 | flbD-up-F | ccattcgccattcaggctgCGGAGAATACCATACATACCAGCG |  |
| 28 | flbD-up-R | gagccgaaccatccaaaaagacaatCAGAGACACACACACGCACACACACA |  |
| 29 | flbE-up-F | tcgccattcaggctgGAATTCGGTATTTCTGATCTCGCGGG |  |
| 30 | flbE-up-R | ggcggtcagcactggcagtaTGGGACTCGCATTGTCCGCCGG |  |
| 31 | flbA-down-F | ccccgtcggccatctactactgaATCCTACAATCAAATAAGACATATAA |  |
| 32 | flbA-down-R | cgcaaaccgcctctcGAATTCGCCACACTCGCTGTACCCTG |  |
| 33 | flbB-down-F | ccccgtcggccatctactactgaATCCTACAATCAAATAAGACATATAA |  |
| 34 | flbB-down-R | cgcaaaccgcctctcGAATTCTCTCACCCACCGCCCCAAAT |  |
| 35 | flbC-down-F | cccagggatatctttgcgccgtGCCGGGTTCCTTGTAGACTGA |  |
| 36 | flbC-down-R | cgcaaaccgcctctcGAATTCCGCAGCGTCGACACAAAACAC |  |
| 37 | flbD-down-F | cccgcgtgtgtctacacttgtgtATTGTCTTTTTGGATGGTTCGGCTC |  |
| 38 | flbD-down-R | cgcaaaccgcctctcTTCCCTCCTTGACCTGCTCGTG |  |
| 39 | flbE-down-F | ccatactgccagtgctgaccgccTTTGGGTTTGTGCATGTGATTTC |  |
| 40 | flbE-down-R | cgcaaaccgcctctcGAATTCGGTATCGGCTGCACGGGTCT |  |
| 41 | flbA-V-F | ACCTCTGCTCCTCTCTCTCTAT |  |
| 42 | flbA-V-R | CTCGCATATTCAATCAGGTAGAT |  |
| 43 | flbB-V-F | ACCCACGGACAGTAGACGGC |  |
| 44 | flbB-V-R | TCTCACCCACCGCCCCAAAT |  |
| 45 | flbC-V-F | ACACTGGCGCCGTTTCGAAG |  |
| 46 | flbC-V-R | CGCAGCGTCGACACAAAACAC |  |
| 47 | flbD-V-F | AGCGGGTACTGAATGGGGC |  |
| 48 | flbD-V-R | CAAGACCCCATCCAGAATGG |  |
| 49 | flbE-V-F | GGTATTTCTGATCTCGCGGGGC |  |
| 50 | flbE-V-R | GGTATCGGCTGCACGGGTCT |  |
| 51 | flbA-UP-C-R | taaccgcgaggacgtctgatgcATCTTGGCGGGAGATACGGTAGGTCC |  |
| 52 | flbB-UP-C-R | ccaccatttattgaagtcatGAGGACGGAGGCGGGAAGAA |  |
| 53 | flbC-UP-C-R | ttctcaatgaccattgtcaTAGGTCAAGAAGGGAATTGCGCGG |  |
| 54 | flbD-UP-C-R | tccacgacgatgagttggagcCATTGCTGAAGTAGTTGGCAAGGGAAA |  |
| 55 | flbE-UP-C-R | gccgtagagcatgtatacgggcatGACTCGCATTGTCCGCCGG |  |
| 56 | flbA-F | ATGCATCAGACGTCCTCGCGGTTA |  |
| 57 | flbA-R | tgcatgacacacatgcTGCCCCTATCATGACCGTGCTGAGCGGCTCGC |  |
| 58 | flbB-F | ATGACTTCTATAAATGGTGGTCCG |  |
| 59 | flbB-R | tattattattgttattgTTATTATATGTCTTATTTGATTGTAGGAT |  |
| 60 | flbC-F | ATGACAATGGTCATTGAGAACCAG |  |
| 61 | flbC-R | gactgaaatgaagagtggcTTGATTTACTCTTCGTCTTCGCCCGAAGC |  |
| 62 | flbD-F | ATGGCTCCAACTCATCGTCGTGGA |  |
| 63 | flbD-R | gcaatgggagctatcgtcatTTAGTTGAGCAAGTTCTGAAGACC |  |
| 64 | flbE-F | ATGCCCGTATACATGCTCTACGGC |  |
| 65 | flbE-R | tatagccatagccatgaccaTCAAGCCGTTCGCCTAGCAAACAA |  |
| 66 | gRNA-flbA-T-FC | ATTTCCAAAAGGGAGAAAGAgacgagcttactcgtttcg |  |
| 67 | gRNA-flbB-T-FC | CGTCGGCCATCTACTACTGAgacgagcttactcgtttcg |  |
| 68 | gRNA-flbC-T-FC | CACGGCGCAAAGATATCCCTgacgagcttactcgtttcg |  |
| 69 | gRNA-flbD-P-FC | ACACAAGTGTAGACACACGCgacgagcttactcgtttcg |  |
| 70 | gRNA-flbE-P-FC | GGCGGTCAGCACTGGCAGTAgttttagagctagaaatagcaag | |
| 71 | gRNA-flbA-T-FC | TCTTTCTCCCTTTTGGAAATgttttagagctagaaatagcaag |  |
| 72 | gRNA-flbB-T-FC | CGTCGGCCATCTACTACTGAgttttagagctagaaatagcaag |  |
| 73 | gRNA-flbC-T-FC | AGGGATATCTTTGCGCCGTGgttttagagctagaaatagcaag |  |
| 74 | gRNA-flbD-T-FC | GCGTGTGTCTACACTTGTGTgttttagagctagaaatagcaag | |
| 75 | gRNA-flbE-T-FC | ACACAAGTGTAGACACACGCgttttagagctagaaatagcaag |  |
| 76 | flbA-CV-UP-F | CTCTCTCTCGCTCGCTCGCT |  |
| 77 | flbA-CV-UP-R | AGGTAGTGGTTGTTGTGGTTGTAA |  |
| 78 | flbA-CV-DOWN-F | TGAGACCGTTCTCATTGCCGAGC |  |
| 79 | flbA-CV-DOWN-R | GCCACACTCGCTGTACCCTG |  |
| 80 | flbB-CV-UP-F | ATACACACAAGAGATCTCGGCGGC |  |
| 81 | flbB-CV-UP-R | GAGACACTGAAGCGCCATGATGG |  |
| 82 | flbB-CV-DOWN-F | CCTGGCGACGCTAGACCAG |  |
| 83 | flbB-CV-DOWN-R | CTCTGGCGTTGAACCCCATAGGA |  |
| 84 | flbC-CV-UP-F | CTCAAACTTCGTCCCATTCCACTCC |  |
| 85 | flbC-CV-UP-R | GTTGCGATGCGTCTCAGGATCG |  |
| 86 | flbC-CV-DOWN-F | GCCGTCCAACACCCAGAAGAAG |  |
| 87 | flbC-CV-DOWN-R | TGGGTGTCTTGTATGCTCCAAACAAG |  |
| 88 | flbD-CV-UP-F | AAGAGTCAGAAAATCGGAAAACAG |  |
| 89 | flbD-CV-UP-R | TTCATGCTGCCGTTCCACCA |  |
| 90 | flbD-CV-DOWN-F | GTCTTCAGAACTTGCTCAACTA |  |
| 91 | flbD-CV-DOWN-R | AACCAGTAATTGCACTGTCAC |  |
| 92 | flbE-CV-UP-F | CATTGTTCCCACCGACTGCTCG |  |
| 93 | flbE-CV-UP-R | GATGACTTTCCCACCGACGTAGG |  |
| 94 | flbE-CV-DOWN-F | ACGTCGGTGGGAAAGTCATCTCC |  |
| 95 | flbE-CV-DOWN-R | ACACCCAAACCCGCTTTCTGG |  |
| 96 | pUC19-F | GAGAGGCGGTTTGCGTATT |  |
| 97 | pUC19-R | CAGCCTGAATGGCGAATGG |  |

**
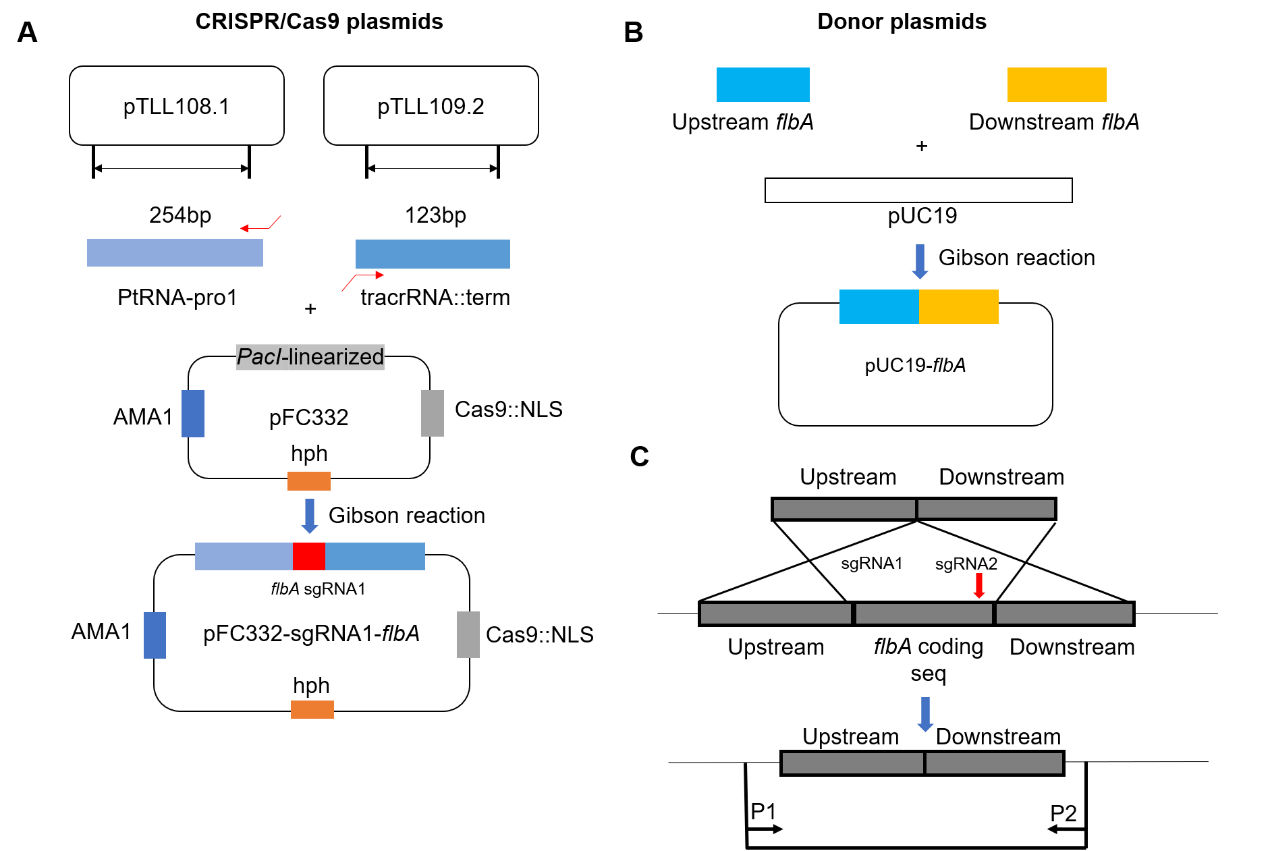
Supplemental Figure 1.** Construction of plasmids used to inactivate *flbA*, *flbB*, *flbC*, *flbD* and *flbE* in reference strain MA234.1 using *flbA* (A, B) and sgRNA1 (A) as an example and PCR verification strategy of the knockout strains using primers P1 and P2 (C).

**
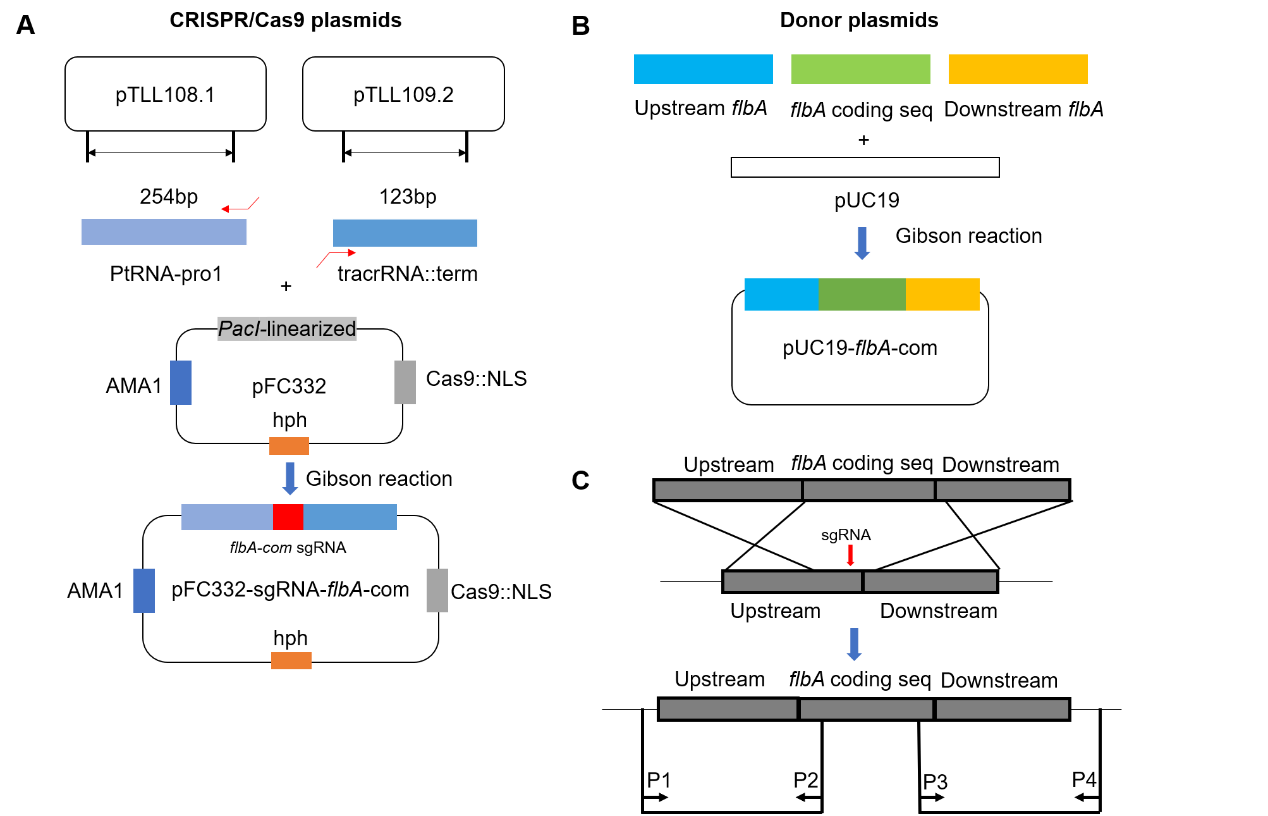
Supplemental Figure 2.** Construction of plasmids used for reintroduction of the *flb* genes in the Δ*flbA*, Δ*flbB*, Δ*flbC*, Δ*flbD* and Δ*flbE* deletion strains. The construct of *flbA* (A, B) and PCR verification of its reintroduction (C) is used as an example.
